# Abnormal Distracter Processing in Adults with Attention-Deficit-Hyperactivity Disorder

**DOI:** 10.1371/journal.pone.0033691

**Published:** 2012-03-22

**Authors:** Frank Marzinzik, Michael Wahl, Doris Krüger, Laura Gentschow, Michael Colla, Fabian Klostermann

**Affiliations:** 1 Department of Neurology, Campus Benjamin Franklin, Charité–University Medicine, Berlin, Germany; 2 Department of Psychiatry, Campus Benjamin Franklin, Charité–University Medicine, Berlin, Germany; Tokyo Metropolitan Institute of Medical Science, Japan

## Abstract

**Background:**

Subjects with Attention-Deficit Hyperactivity Disorder (ADHD) are overdistractible by stimuli out of the intended focus of attention. This control deficit could be due to primarily reduced attentional capacities or, e. g., to overshooting orienting to unexpected events. Here, we aimed at identifying disease-related abnormalities of novelty processing and, therefore, studied event-related potentials (ERP) to respective stimuli in adult ADHD patients compared to healthy subjects.

**Methods:**

Fifteen unmedicated subjects with ADHD and fifteen matched controls engaged in a visual oddball task (OT) under simultaneous EEG recordings. A target stimulus, upon which a motor response was required, and non-target stimuli, which did not demand a specific reaction, were presented in random order. Target and most non-target stimuli were presented repeatedly, but some non-target stimuli occurred only once (‘novels’). These unique stimuli were either ‘relative novels’ with which a meaning could be associated, or ‘complete novels’, if no association was available.

**Results:**

In frontal recordings, a positive component with a peak latency of some 400 ms became maximal after novels. In healthy subjects, this novelty-P3 (or ‘orienting response’) was of higher magnitude after complete than after relative novels, in contrast to the patients with an undifferentially high frontal responsivity. Instead, ADHD patients tended to smaller centro-parietal P3 responses after target signals and, on a behavioural level, responded slower than controls.

**Conclusion:**

The results demonstrate abnormal novelty processing in adult subjects with ADHD. In controls, the ERP pattern indicates that allocation of meaning modulates the processing of new stimuli. However, in ADHD such a modulation was not prevalent. Instead, also familiar, only context-wise new stimuli were treated as complete novels. We propose that disturbed semantic processing of new stimuli resembles a mechanism for excessive orienting to commonly negligible stimuli in ADHD.

## Introduction

Subjects with attention deficit/hyperactivity disorder (ADHD) have difficulties to control attentional targets [1]–[3], apparently corresponding to increased distractibility by extraneous stimuli. However, it remains to be settled whether this decreased concentration on one subject is due to primarily reduced attentional resources or to increased distracter processing, possibly resulting in an arbitrary invasion of stimuli into the focus attention.

Correlates of target and distracter processing can be studied on the basis of event-related potentials (ERP), particularly so called ‘P3’ (for P300) components, peaking between 300 and 600 ms after eliciting stimuli [4]–[9]. The parietal P3 is mostly studied in oddball paradigms. It is of larger magnitude after target signals, instructive for the task demand, than after irrelevant non-target signals, and its expression mostly implies sustained attention in goal-directed behaviour. Correspondingly, it has been found reduced in ADHD patients [10]–[12]. The frontal ‘novelty P3’ reflects newness of stimuli rather than their task relevance. It mirrors neurophysiological processes underlying orienting reactions to stimuli conquering the focus of attention and, consequently, has been studied in ADHD as an index of distracter processing. However, both disease-related enhancement and reduction of the novelty P3 has been reported [11], [13]–[16].

One factor for this variability might be that semantic stimulus properties, influencing the expression of novelty-related ERP [17]–[20], have not been controlled in according studies. Therefore, we were interested in whether the availability of connotations for novel events distinctly affected healthy subjects and patients with ADHD. We expected that stimuli which are virtually new were differentiated from stimuli which are unique in the ongoing context, but principally known. The rationale for this assumption was that, from a behavioural perspective, it is crucial to spend attention to information with unknown implications, whereas it appears advantageous to avoid shifts from the sustained focus of attention if putative distracters can be categorised as task-irrelevant. A potential dysfunction of such stimulus weighing in ADHD would increase orienting reactions to indeed new, but otherwise hardly distractive events. Accordingly, we hypothesised that whether stimulus connotations were available or not should be a factor for the expression of the novelty P3 in healthy subjects, but not in patients with ADHD.

In order to test this hypothesis, we analysed the ERP of patients and matched controls in a modified oddball task (OT). Next to the typical repetition of non-target and target stimuli, some non-target stimuli were only presented once. These stimuli belonged to two subclasses in that the participants could either associate a meaning with them or not. On this basis, it could, first, be analysed if novelty-related ERP were modulated by the semantic familiarity of eliciting stimuli and, second, if such modulation was abnormal in ADHD subjects, indicative of an impairment of implicit distracter evaluation in this condition.

## Methods

### Participants

Fifteen unmedicated adult subjects with ADHD (9 females, 6 males; 32,4±7,2 years) were recruited from the outpatient clinic of the Department of Psychiatry of the Charité, Campus Benjamin Franklin (CBF). All participants gave written informed consent to the study protocol, approved by the Ethics Committee of the Charité.

Clinical assessment of the patients was conducted according to the diagnostic guidelines for ADHD in adulthood as outlined by the expert consensus of the German Society for Psychiatry, Psychotherapy and Neurology [21]. The cornerstone of this protocol was the semi-structured Conners' Adult ADHD Diagnostic Interview for DSM-IV (CAADID).

Several standardized self-report and collateral informant rating scales designed to quantify ADHD symptoms both currently and retrospectively were also employed. Childhood ADHD symptoms were self-rated with the short-version of the Wender Utah Rating Scale (WURS-k) [22]–[23] including 25 items on a 5-point Likert-scale (“not at all” to “severe”, cut-off score 30, maximum score 84). Severity of adulthood ADHD symptoms was self-rated with the ADHD-Checklist [24] including 18 items on a 3-point Likert-scale corresponding to the diagnostic criteria of DSM-IV (ranging from “not at all” to “severe”, maximum score 36). Current comorbidities with Axis-I-disorders and lifetime history of psychotic, bipolar and substance abuse disorder were excluded using the SCID-I [25] and the current score for the Beck-Depression-Inventory (BDI) was raised [26]. A diagnosis was given to individuals fulfilling DSM-IV criteria for childhood ADHD only under consensus of a graduate level clinical psychologist and a board certified psychiatrist after careful review of the data acquired via this assessment protocol.

Additionally, fifteen age-matched and healthy control subjects (10 females, 5 males; 29,9±7,7 years) participated in the study. They had to meet the same exclusion criteria and did not suffer from ADHD, as determined by DSM-IV. The exploration/examination of participants was carried out by clinical psychologists and psychiatrists as detailed above. An overview of the study cohorts is provided in Table 1.

**Table 1:** Study Cohorts.

	*controls*	*patients*
*number (m/f)*	15 (6/9)	15 (5/10)
*age*	32.4±7.2	29.9±7.7
*education (years)*	12.07±1.38	12.7±0.79
*WURS-k*	8.2±2.0	40.6±13.5
*ADHD-checklist*	4.2±3.1	26.9±5.1
*BDI*	7.6±2.7	7.0±2.8

Demographic data and clinical specifics of patients and controls as assessed by the Wender Utah Rating Scale (WURS-K), ADHD-checklist, Beck Depression Inventory (BDI).

### Experimental procedure

**Figure 1:**
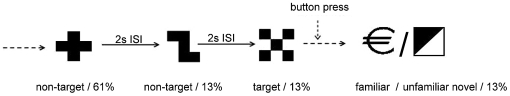
Modified oddball task. Altogether 460 target, non-target and novel (non-target) symbols were presented to each subject in the current modified oddball task. Meaning-wise, novels were either familiar (relative novel) or non-familiar (complete novel). The symbols appeared at intervals of 2 seconds in randomised order.

Experimental procedures were performed in the Department of Neurology, CBF. Patients and healthy controls engaged in a modified oddball task, comprising 460 visual stimuli with presentation time of 150 ms at interstimulus intervals of 2000 ms. All stimuli appeared within a quadratic frame of 6×6 cm^2^ in the middle of a 15″ computer screen, participants sitting at a distance of 1.5 m. An x-like stimulus with an occurrence probability of 13% was defined as target upon which a right index finger button press had to be carried out as fast as possible (Figure 1). Non-targets occurred at two probabilities, at 13% (z-like shape) and 61%, (plus sign). The remaining 13% of stimuli were non-target ‘novels’, each presented once only during the experiment. After task completion, the participants had to categorise the novels as to (i) whether they could associate a meaning with the respective stimulus (in the following labelled as ‘familiar novel’) or (ii) whether nothing could be associated with it (in the following labelled as ‘non-familiar novel’). The selection of novels was based on a pilot study with 42 participants who had classified 100 stimuli (from free fonts for Microsoft Word) with respect to this criterion. For the present paradigm each thirty stimuli with which most of the 42 subjects could/could not associate a meaning were used (i. e., the most familiar and most non-familiar novel stimuli).

With respect to behavioral task performance, reaction times and accuracy were determined (assessing omissions of target responses as well as responses to non-target stimuli).

### Analysis

For ERP analysis, electroencephalographic recordings were performed from 20 scalp positions over frontal (F7, F3, Fz, F4, F8), fronto-central (FC7, FC3, FCz, FC4, FC8), central (C7, C3, Cz, C4, C8) and parietal sites (P7, P3, Pz, P4, P8). Peristimulus segments were averaged from the EEG, filtered from 0.05–20 Hz, for each stimulus class, i. e. target stimuli as well as frequent, rare, familiar novel and non-familiar novel non-target stimuli (epochs from 150 ms before to 1500 ms after stimulus presentation). Trials with eye movement or blink artefacts were excluded from further analysis. Peaks of P3 components were defined as the most positive deflection within a time window from 300 ms to 600 ms after stimulus presentation. Amplitudes were determined with respect to the baseline, covering 150 ms before stimulus presentation.

For statistical analysis, separate ANOVAs were run for each region. Since the primary aim was to explore the group-specific responsivity to familiar versus non-familiar novel stimuli, the assessment of oddball and novelty effects per region served to confirm that these well studied factors produced largest effects in the expected recordings. After this data check, familiarity effects on the regionally typical components were explored. The details of these ANOVAs are provided in the according paragraphs of the following chapter.

## Results

### Categorisation of novel stimuli

**Figure 2:**
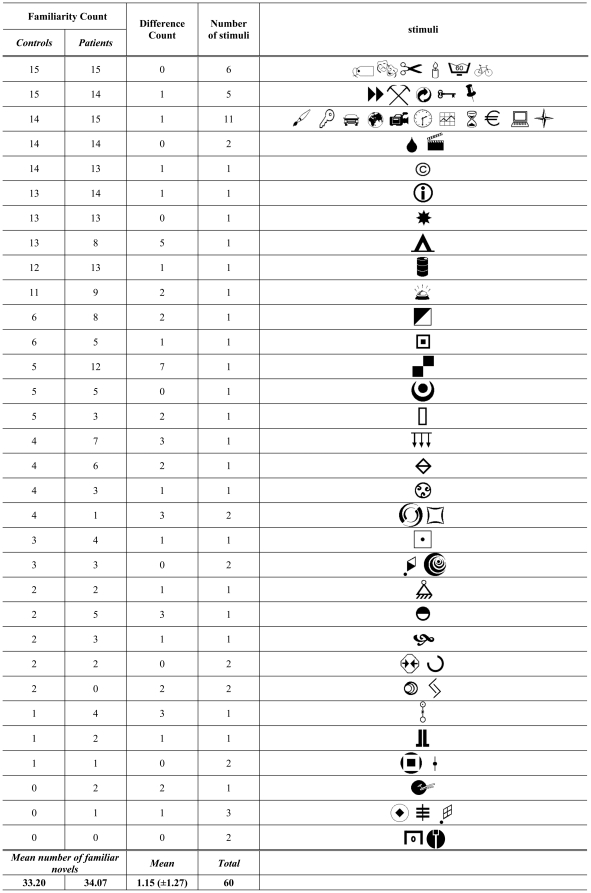
Categorisation of novel stimuli. Categorisation of the stimuli strongly overlapped between groups. This can be deduced from the present description, in which the sixty novel stimuli were ordered according to semantic familiarity scores in the control cohort: First and second columns provide scores per group, the highest familiarity being 15 (meaning that all group members could associate a meaning with the given stimulus), the lowest 0 (meaning that none of the group members could associate a meaning with the given stimulus). In the third column, between-group differences for stimuli with the indicated rating constellation are presented, expressed as the rating score of controls minus that of patients. In the last column, the number of stimuli with the between-group rating-constellation, specified in the respective row, is indicated.

Controls categorised 33.2/26.8 of the 60 novel stimuli as familiar/non-familiar. The according numbers for the patients were 34.1/25.9. Between the groups, the number of discrepant ratings of stimulus familiarity was generally low with a difference of 1.15±1.27 per symbol, 0 meaning that all ratings corresponded between controls and patients and 15 indicating that all judgments in the ADHD group differed from the judgements of healthy controls. No statistical difference was identified between patients and healthy controls with respect to familiarity ratings of the stimuli (p = .87 by planned two-sided paired t-test). The details of the stimulus categorisation are provided in Figure 2.

### Behavioral data

The error rates were calculated as the percentage of incorrect reactions referenced to the required reactions (omission of targets reaction, responses to non-targets). For statistical analysis, a two-way-ANOVA with the within-subject factor *task condition* (4 levels: target, frequent non-target, rare non-target, novel non-target) and the between-subject factor *group* (2 levels: controls/patients) was run. Post-hoc comparisons were calculated with Newman-Keuls tests. For all ANOVAs, data were Greenhouse-Geisser corrected.

A significant interaction group x task condition was identified (F[2,56] = 6.23, p<.01). Post-hoc testing revealed that the omission rate of target responses was significantly higher in patients (2.7±4.0%) than in controls (0.1±0.4%; p<.01). The groups did not differ with respect to false responses to rare, frequent and novel non-targets.

With respect to reaction time to target responses, a one-way-ANOVA with the between-subject factor *group* was performed (2 levels: controls/patients; here task condition was not an additional test factor since reactions were not demanded to any other stimulus category). This showed that patients responded significantly slower (501±92 ms) than controls (415±48 ms; F[1,28] = 10.24, p<.01).

### Event-related potentials (ERP)

**Figure 3:**
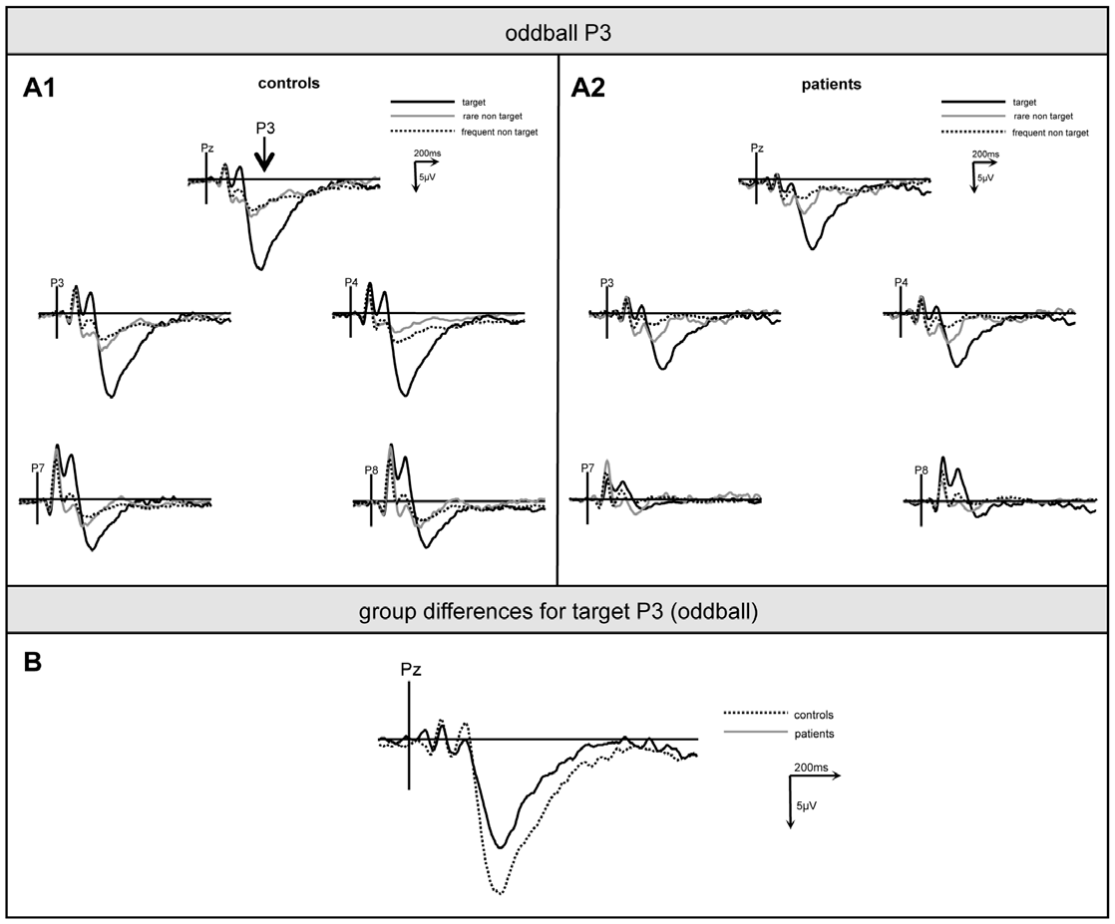
Oddball P3. Grand-average of ERP from parietal electrodes (P8, P4, Pz, P3, P7) upon targets (bold line), rare (thin line) and frequent non-targets (dotted line) in controls (A1) and patients (A2). (B) shows target-P3 differences between controls (dotted line) and patients (bold line).

In order to test oddball effects, a three-way-ANOVA with the between-subject factor *group* (levels: controls/patients) and the within-subject factors *task condition* (levels: target, frequent non-target, rare non-target) and *electrode* (5 levels). To explore if the regional distribution of task effects conformed to previous findings for the ‘oddball P3’, this analysis was separately run for frontal (F7, F3, Fz, F4, F8), fronto-central (FC7, FC3, FCz, FC4, FC8), central (C7, C3, Cz, C4, C8) and parietal ERP (P7, P3, Pz, P4, P8). In line with the literature [9], [27], the factor *task condition* was strongest in parietal recordings (F[2,56] = 56.02, p<.001). Here, post-hoc tests proved potentials to be larger upon target stimuli (14.1±9.7 µV) than upon rare (7.4±3.9 µV) and frequent non-target (4.2±3.7 µV; both comparisons: p<.001); further, rare non-target stimuli elicited larger components than frequent non-target stimuli (p<.001, see Figure 3). Besides, there was a strong trend (F[1,28] = 4.0, p = .055) to larger P3 in controls (10.1±8.2 µV) than in patients (7.1±8.2 µV), independent from the target or non-target status of the eliciting event.

Novelty effects were analysed with the same ANOVA design (between-subject factor *group* [levels: controls/patients], within-subject factors *task condition* [levels: frequent non-target, rare non-target, novel non-target] and *electrode* [5 levels], separately for frontal, fronto-central, central and parietal ERP). As expected from the literature [9], [27], *Task condition* was a main factor with the highest effect in frontal recordings (F[2,56] = 12.06, p<.001). Post hoc tests revealed that novel non-targets elicited larger ERP (6.8±5.4 µV) than rare (5.4±3.1 µV; p<.05) and frequent non-targets (3.84±2.7 µV, p<.001).

**Figure 4:**
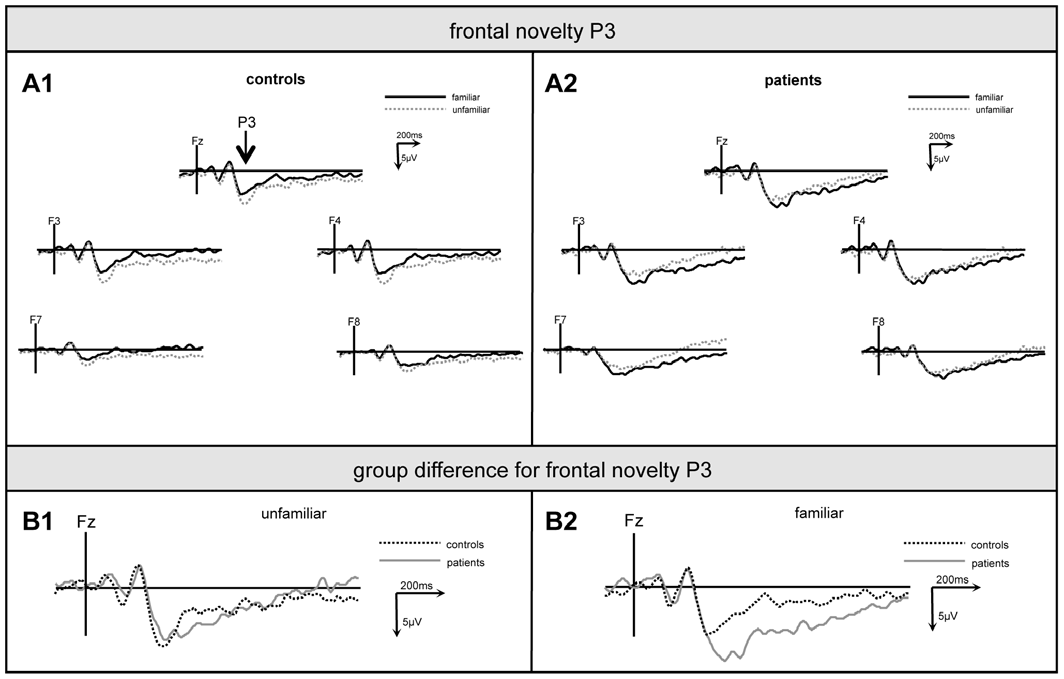
Familiarity effect on frontal P3. Grand-average of ERP from frontal electrodes (F8, F4, Fz, F3, F7) upon familiar (bold line) and unfamiliar novels (dotted line) in controls (A1) and patients (A2). B1 shows ERP-differences for familiar, B2 for unfamiliar novels between controls (dotted line) and ADHD-patients (bold line).

**Figure 5:**
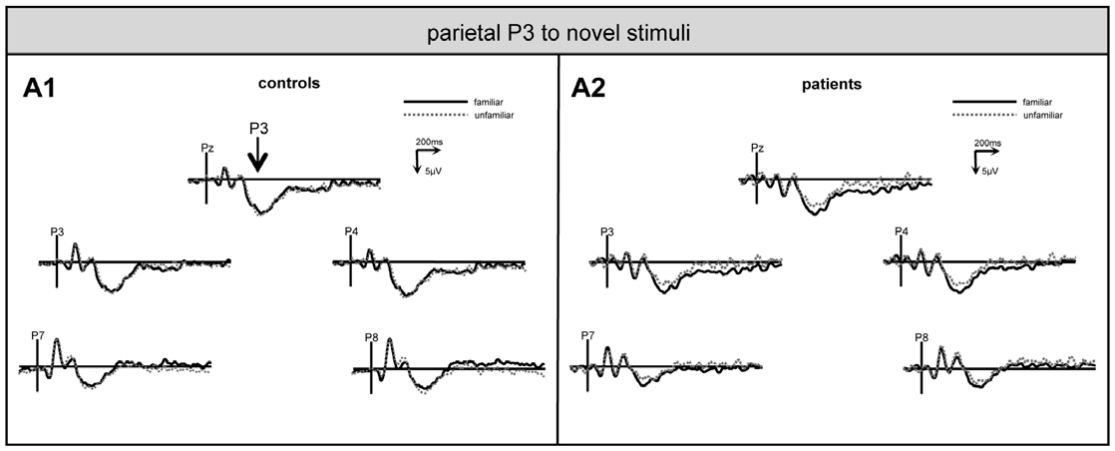
Familiarity effect on parietal P3. Grand-average of ERP from parietal electrodes (P8, P4, Pz, P3, P7) upon familiar (bold line) and unfamiliar novels (dotted line) in controls (A1) and patients (A2).

**Figure 6:**
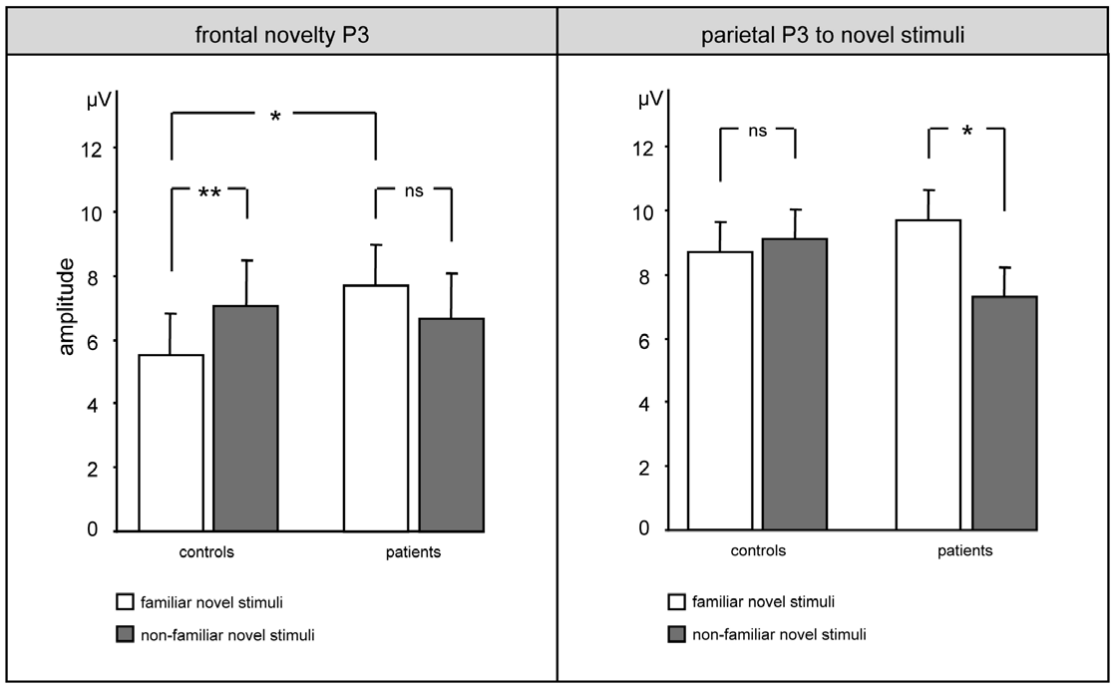
Statistical comparison of novelty P3. Columns indicate mean novelty-P3 amplitude to familiar and unfamiliar novels, bars show the respective standard errors of mean (ns = not significant, * indicates P<.05).

Finally, the familiarity effect in novelty processing was analysed in this ANOVA design (between-subject factor *group* [levels: controls/patients], within-subject factors *task condition* [levels: familiar/non-familiar novel non-target] and *electrode* [5 levels]). Interactions *task condition x group* were identified at frontal and parietal recording positions (frontal: F[1,28] = 12.34, p<.001/parietal: F[1,28] = 4.71, p<.05). For the frontal ANOVA, the post-hoc analysis revealed that this interaction was due to the fact that ERP upon non-familiar novels (7.1±6.6 µV) were larger than upon familiar novels (5.6±5.5 µV) in controls (p<.05), but that this was not the case in patients (non-familiar novels: 6.6±4.9 µV; familiar novels: 7.7±5.1 µV) and that, further, frontal ERP upon familiar novels were larger in patients than in controls (p<.05, see Figure 4). In turn, for the the parietal ANOVA the post-hoc analysis revealed that the interaction relied on larger ERP upon familiar than non-familiar novels in the patients only (familiar novels: 9.7±4.1 µV; non-familiar novels: 7.3±3.9 µV; p<.05), whereas no significant difference was obtained in controls (familiar novels: 8.7±5.1 µV; non-familiar novels: 9.1±5.2 µV; see Figure 5). A summary of these results is provided by Figure 6.

Parallel analyses of components prior to the P3 components did not yield significant results. ERP latencies did not differ between groups (Table 2).

**Table 2:** ERP peak latencies.

	novelty-P3		oddball-P3b		
	familiar	unfamiliar	target	frequent non-target	rare non-target
**controls**	439.8±55.7	441.4±45.9	471.7±46.3	417.6±57.0	419.6±53.8
**patients**	473.3±65.7	477.3±60.5	476.9±46.6	424.2±43.9	412.9±45.9

Mean peak-latencies in milliseconds (± SD) for the frontal novelty-P3 and the centro-parietal oddball-P3 for controls and patients.

## Discussion

Healthy subjects expressed distinct frontal event-related potentials (ERP) to semantically familiar versus non-familiar stimuli, presented as unique non-target signals (novels) in an oddball task. In patients with ADHD this was not the case, but ERPs to the mentioned signal categories differed in parietal recordings. However, the ‘posteriorised’ differentiation of novels in the patients appeared opposite to the distinction in controls.

For the interpretation of these findings, some concepts of the respective ERP shall be briefly recalled. In the control group, the mentioned ERP distinction refers to the novelty P3, in frontal recordings peaking about 400 to 500 ms after unique stimuli interspersed between repeatedly presented events. This component has been allocated to networks, comprising cingulate, prefrontal, orbitofrontal and temporal sources [5], [20], [27]–[33]. According to its specificity in relation to stimulus newness and for its occurrence independent from controlled attentional targets, it is considered an electrophysiological correlate of the brain's orienting response, automatically adapting behaviour to unpredictable ambient changes [34]–[36]. In healthy subjects this component was of larger magnitude after non-familiar than familiar novels, suggesting that its expression is not exclusively influenced by contextual stimulus newness, but also by signal content. Functionally, this appears reasonable, because semantic analysis of behaviourally irrelevant, new stimuli could prevent the organism from unnecessary shifts from the sustained focus of attention. In the same vein, each stimulus for which semantic information is unavailable should draw attention on itself for its unpredictable implications, compatible with larger frontal P3 upon non-familiar than familiar novels [9], [37], [38]. Previous findings indeed point to semantic analysis as part of novelty processing [17], [18]. For example, functional imaging has demonstrated activation of the inferior frontal gyrus upon presentation of respective stimuli, interpreted as the ‘extraction of stimulus meaning, thereby enabling one to determine the significance of the environmental perturbation and take appropriate goal-directed action’ [34]. Further, concerning frontal P3 potentials in particular, amplitudes were found reduced in patients with hippocampal lesions, which has been proposed to rely on deficient automatic recall of novel-related information [39].

In this view, the frontal P3 distinction in controls reflects compound actvities from a network specialised in the detection of both stimulus newness and meaning. Accordingly, the patients' generally high frontal responsivity to novels, whether familiar or not, could be interpreted as a reflection of enhanced orienting to stimuli which, after normal semantic analysis, would eventually be less distractive.

However, in the ADHD group the category of novels influenced the expression of parietal P3 responses. This posterior component from cingulate and temporoparietal generators [40]–[44] reflects the salience and intentionally ascribed importance of eliciting events. Becoming maximal after target stimuli in the conscious focus of attention [45]–[47], it probably reflects the matching of incoming with task-related target signals [9], [48] and, thus, is rather related to controlled operations than the frontal novelty P3. With respect to the present data, it is noteworthy that factors of enhanced stimulus salience normally induce increases of both frontal and parietal P3. However, in controls only the frontal, but not the parietal component differed between novels, categorised as familiar versus unfamiliar, whereas in ADHD patients the opposite was the case. Further, the change direction of frontal and parietal P3 in controls and patients was inverse, inconsistent with a parallel modulation by a categorical salience difference between familiar and unfamiliar novels. Thus although an influence of uncontrolled attributes determining stimulus salience on the ERP distinction between controls and healthy subjects is theoretically possible, an alternative explanation of the results appears more likely.

Frontal and parietal P3 mirror tightly linked processes in attentional control, conceived as the alignment of environmental change (frontal P3) with ongoing behavioural demands (parietal P3) [49]–[51]. Thus, the shift from frontal to parietal novelty distinction in ADHD seems to indicate a disequilibrium, for example, in that deficient automatic content analysis of stimuli facilitates decreased resistance of the sustained focus of attention against irrelevant information. With this view in which abnormal frontal-parietal informational flow [49]–[51] results in an impairment of appropriate processing of task-relevant stimuli, also the relatively small parietal P3 to target stimuli in the patients fits in – a well known result from children with ADHD [10]–[12].

With respect to the concept of ADHD as a disorder of frontal inhibition [52]–[56], it is of note that P3 potentials are indeed thought to mirror the phasic suppression of ongoing operations in support of processing the eliciting, e. g., new event [9], [50]. However, the abnormal ERP pattern in the patients does not simply point to general hypoinhibition in ADHD [57]–[60], but rather to imbalanced inhibitory processing prevailing in this condition.

On a behavioural level, ADHD patients and healthy subjects categorised the familiarity of stimuli almost identically, a result which comes as no surprise, given that subjects completed this debriefing procedure without any time limit so that of putative correlates to ERP differences were not expected on this level. However, patients differed from controls with respect to the proper task performance. Resembling findings in children and adolescents with ADHD [61]–[62], the adult patients showed increased response latency and inaccuracy. In this regard, it can – by analogy to the above notions on frontal and parietal P3 – be presumed that in ADHD novel information is undifferentially processed at the expense of attention demanding, task-related operations and, therefore, of swift and precise responding to target stimuli.

In conclusion, we propose that in ADHD the automatic recall of semantic information on new stimuli is deficient, reflected by an undifferentiated generation of high amplitude novelty-P3 potentials. In this concept, overshooting categorisation of stimuli as distracters results in excessive orienting responses to normally negligible events and shifts ADHD patients away from the sustained focus of attention and ongoing behavioural plans.

## References

[1] Adams R, Finn P, Moes E, Flannery K, Rizzo AS (2009). Distractibility in Attention/Deficit/Hyperactivity Disorder (ADHD): the virtual reality classroom.. Child Neuropsychol.

[2] Dopheide JA, Pliszka SR (2009). Attention-deficit-hyperactivity disorder: an update.. Pharmacotherapy.

[3] Moss SB, Nair R, Vallarino A, Wang S (2007). Attention deficit/hyperactivity disorder in adults.. Prim Care.

[4] Cycowicz YM, Friedman D (2004). The old switcheroo: when target environmental sounds elicit a novelty P3.. Clin Neurophysiol.

[5] Debener S, Makeig S, Delorme A, Engel AK (2005). What is novel in the novelty oddball paradigm? Functional significance of the novelty P3 event-related potential as revealed by independent component analysis.. Brain Res.

[6] Daffner KR, Scinto LF, Calvo V, Faust R, Mesulam MM (2000). The influence of stimulus deviance on electrophysiologic and behavioral responses to novel events.. J Cogn Neurosci.

[7] Daffner KR, Ryan KK, Williams DM, Budson AE, Rentz DM (2005). Age-related differences in novelty and target processing among cognitively high performing adults.. Neurobiol Aging.

[8] Friedman D, Kazmerski VA, Cycowicz YM (1998). Effects of aging on the novelty P3 during attend and ignore oddball tasks.. Psychophysiology.

[9] Polich J (2007). Updating P300: an integrative theory of P3a and P3b.. Clin Neurophysiol.

[10] Holcomb PJ, Ackerman PT, Dykman RA (1985). Cognitive event-related brain potentials in children with attention and reading deficits.. Psychophysiology.

[11] Kemner C, Verbaten MN, Koelega HS, Buitelaar JK, van der Gaag RJ (1996). Event-related brain potentials in children with attention-deficit and hyperactivity disorder: effects of stimulus deviancy and task relevance in the visual and auditory modality.. Biol Psychiatry.

[12] Satterfield JH, Schell AM, Nicholas TW, Satterfield BT, Freese TE (1990). Ontogeny of selective attention effects on event-related potentials in attention-deficit hyperactivity disorder and normal boys.. Biol Psychiatry.

[13] Holcomb PJ, Ackerman PT, Dykman RA (1986). Auditory event-related potentials in attention and reading disabled boys.. Int J Psychophysiol.

[14] Gumenyuk V, Korzyukov O, Alho K, Escera C, Näätänen R (2004). Effects of auditory distraction on electrophysiological brain activity and performance in children aged 8–13 years.. Psychophysiology.

[15] Gumenyuk V, Korzyukov O, Alho K, Escera C, Schröger E (2001). Brain activity index of distractibility in normal school-age children.. Neurosci Lett.

[16] Gumenyuk V, Korzyukov O, Escera C, Hämäläinen M, Huotilainen M (2005). Electrophysiological evidence of enhanced distractibility in ADHD children.. Neurosci Lett.

[17] Escera C, Yago E, Corral MJ, Corbera S, Nuñez MI (2003). Attention capture by auditory significant stimuli: semantic analysis follows attention switching.. Eur J Neurosci.

[18] Mecklinger A, Opitz B, Friederici AD (1997). Semantic aspects of novelty detection in humans.. Neurosci Lett.

[19] Opitz B, Mecklinger A, Friederici AD, von Cramon DY (1999). The functional neuroanatomy of novelty processing: integrating ERP and fMRI results.. Cereb Cortex.

[20] Ranganath C, Paller KA (1999). Frontal brain activity during episodic and semantic retrieval: insights from event-related potentials.. J Cogn Neurosci.

[21] Ebert D, Krause J, Roth-Sackenheim C (2003). ADHD in adulthood-guidelines based on expert consensus with DGPPN support.. Nervenarzt.

[22] Retz-Junginger P, Retz W, Blocher D, Weijers HG, Trott GE (2002). Wender Utah rating scale. The short-version for the assessment of the attention-deficit hyperactivity disorder in adults.. Nervenarzt.

[23] Ward MF, Wender PH, Reimherr FW (1993). The Wender Utah Rating Scale: An aid in the retrospective diagnosis of childhood Attention Deficit Hyperactivity Disorder.. Am J Psychiatry.

[24] Rösler M, Retz W, Retz-Junginger P, Thome J, Supprian T (2004). Tools for the diagnosis of attention-deficit/hyperactivity disorder in adults. Self-rating behaviour questionnaire and diagnostic checklist.. Nervenarzt.

[25] First M, Spitzer R (1997). Structured Clinical Interview for DSM-IV AXIS I Disorders (Clinician Version) SCID-I Administration Booklet.

[26] Beck AT, Ward CH, Mendelson M, Mock J, Erbaugh J (1961). An inventory for measuring depression.. Arch Gen Psychiatry.

[27] Volpe U, Mucci A, Bucci P, Merlotti E, Galderisi S (2007). The cortical generators of P3a and P3b: a LORETA study.. Brain Res Bull.

[28] Friedman D, Cycowicz YM, Gaeta H (2001). The novelty P3: an event-related brain potential (ERP) sign of the brain's evaluation of novelty.. Neurosci Biobehav Rev.

[29] He B, Lian J, Spencer KM, Dien J, Donchin E (2001). A cortical potential imaging analysis of the P300 and novelty P3 components.. Hum Brain Mapp.

[30] Baudena P, Halgren E, Heit G, Clarke JM (1995). Intracerebral potentials to rare target and distractor auditory and visual stimuli. III. Frontal cortex.. Electroencephalogr Clin Neurophysiol.

[31] Brazdil M, Rektor I, Dufek M, Daniel P, Jurak P (1999). The role of frontal and temporal lobes in visual discrimination task-depth ERP studies.. Neurophysiol Clin.

[32] Clark VP, Fannon S, Lai S, Benson R, Bauer L (2000). Responses to rare visual target and distractor stimuli using event-related fMRI.. J Neurophysiol.

[33] Bledowski C, Prvulovic D, Goebel R, Zanella FE, Linden DE (2004). Attentional systems in target and distractor processing: a combined ERP and fMRI study.. Neuroimage.

[34] Friedman D, Goldman R, Stern Y, Brown TR (2009). The brain's orienting response: An event-related functional magnetic resonance imaging investigation.. Hum Brain Mapp.

[35] Lynn R, Eysenck HJ (1966). Attention, Arousal and the Orientation Reaction.. International series of monographs in experimental psychology, Vol. 3.

[36] Pavlov I (1927). Conditioned Reflexes.

[37] Vila J, Guerra P, Muñoz MA, Vico C, Viedma-del Jesús MI (2007). Cardiac defense: from attention to action.. Int J Psychophysiol.

[38] Sokolov EN (1990). The orienting response, and future directions of its development.. Pavlov J Biol Sci.

[39] Knight RT (1996). Contribution of human hippocampal region to novelty detection.. Nature.

[40] Verleger R, Heide W, Butt C, Kompf D (1994). Reduction of P3b in patients with temporo-parietal lesions.. Brain Res Cogn Brain Res.

[41] Soltani M, Knight RT (2000). Neural origins of the P300.. Crit Rev Neurobiol.

[42] He B, Lian J, Spencer KM, Dien J, Donchin E (2001). A cortical potential imaging analysis of the P300 and novelty P3 components.. Hum Brain Mapp.

[43] Goldstein A, Spencer KM, Donchin E (2002). The influence of stimulus deviance and novelty on the P300 and novelty P3.. Psychophysiology.

[44] Dien J, Spencer KM, Donchin E (2003). Localization of the event-related potential novelty response as defined by principal components analysis.. Brain Res Cogn Brain Res.

[45] Johnson R, Donchin E (1978). On how P300 amplitude varies with the utility of the eliciting stimuli.. Electroencephalogr Clin Neurophysiol.

[46] Sutton S, Braren M, Zubin J, John ER (1965). Evoked-potential correlates of stimulus uncertainty.. Science.

[47] Polich J (1990). P300, probability, and interstimulus interval.. Psychophysiology.

[48] Donchin E, Coles MG (1988). Is the P300 component a manifestation of context updating?. Behav Brain Sci.

[49] Soltani M, Knight RT (2000). Neural origins of the P300.. Crit Rev Neurobiol.

[50] Polich J, Comerchero MD (2003). P3a from visual stimuli: typicality, task, and topography.. Brain Topogr.

[51] Knight R, Rohbraugh J, Parasuraman R, Johnson R (1990). Neural mechanisms of event-related potentials from human lesion studies.. Event-related brain potentials: basic issues and applications.

[52] Boonstra AM, Kooij JJ, Oosterlaan J, Sergeant JA, Buitelaar JK (2010). To act or not to act, that's the problem: primarily inhibition difficulties in adult ADHD.. Neuropsychology.

[53] Bush G, Valera EM, Seidman LJ (2005). Functional neuroimaging of attentiondeficit/hyperactivity disorder: a review and suggested future directions.. Biol Psychiatry.

[54] Fallgatter AJ, Ehlis AC, Rösler M, Strik WK, Blocher D (2005). Diminished prefrontal brain function in adults with psychopathology in childhood related to attention deficit hyperactivity disorder.. Psychiatry Res.

[55] Liotti M, Pliszka SR, Perez R, Kothmann D, Woldorff MG (2005). Abnormal brain activity related to performance monitoring and error detection in children with ADHD.. Cortex.

[56] Nigg JT, Butler KM, Huang-Pollock CL, Henderson JM (2002). Inhibitory processes in adults with persistent childhood onset ADHD.. J Consult Clin Psychol.

[57] Depue BE, Burgess GC, Willcutt EG, Ruzic L, Banich MT (2010). Inhibitory control of memory retrieval and motor processing associated with the right lateral prefrontal cortex: evidence from deficits in individuals with ADHD.. Neuropsychologia.

[58] Booth JR, Burman DD, Meyer JR, Lei Z, Trommer BL (2005). Larger deficits in brain networks for response inhibition than for visual selective attention in attention deficit hyperactivity disorder (ADHD).. J Child Psychol Psychiatry.

[59] Solanto MV, Schulz KP, Fan J, Tang CY, Newcorn JH (2009). Event-related fMRI of inhibitory control in the Predominantly. Inattentive and Combined Subtypes of AD/HD.. Neuroimaging.

[60] Zang YF, Jin Z, Weng XC, Zhang L, Zeng YW (2005). Functional MRI in attention-deficit hyperactivity disorder: evidence for hypofrontality.. Brain Dev.

[61] Tamm L, Menon V, Reiss AL (2006). Parietal attentional system aberrations during target detection in adolescents with attention deficit hyperactivity disorder: event-related fMRI evidence.. Am J Psychiatry.

[62] Uebel H, Albrecht B, Asherson P, Börger NA, Butler L (2010). Performance variability, impulsivity errors and the impact of incentives as gender-independent endophenotypes for ADHD.. J Child Psychol Psychiatry.

